# A population-based study on healthcare-seeking behaviour of persons with symptoms of respiratory and gastrointestinal-related infections in Hong Kong

**DOI:** 10.1186/s12889-020-08555-2

**Published:** 2020-03-27

**Authors:** Qiqi Zhang, Shuo Feng, Irene O. L. Wong, Dennis K. M. Ip, Benjamin J. Cowling, Eric H. Y. Lau

**Affiliations:** WHO Collaborating Centre for Infectious Disease Epidemiology and Control, School of Public Health, Li Ka Shing Faculty of Medicine, The University of Hong Kong, Hong Kong Special Administrative Region, China

**Keywords:** Influenza, Influenza-like illness, Healthcare seeking behaviour, Symptom-specific

## Abstract

**Background:**

Studies on healthcare-seeking behaviour usually adopted a patient care perspective, or restricted to specific disease conditions. However, pre-diagnosis symptoms may be more relevant to healthcare-seeking behaviour from a patient perspective. We described healthcare-seeking behaviours by specific symptoms related to respiratory and gastrointestinal-related infections.

**Methods:**

We conducted a longitudinal population-based telephone survey in Hong Kong. We collected data on healthcare-seeking behaviour specific to symptoms of respiratory and gastrointestinal-related infections and also associated demographic factors. We performed descriptive analyses and estimated the proportion of participants who sought medical consultation, types of services utilized and duration from symptom onset to healthcare seeking, by different age groups. Post-stratification was used to compensate non-response and multiple imputation to handle missing and right-censored data.

**Results:**

We recruited 2564 participants who reported a total of 4370 illness episodes and 7914 symptoms. Fatigue was the most frequently reported symptom, followed by headache and runny nose, with 30-day incidence rate of 9.1, 7.7, and 7.7% respectively. 78% of the participants who had fever sought medical consultation, followed by those with rash (60%) and shortness of breath (58%). Older adults (aged ≥55y) who had symptoms including fever, sore throat, and headache had a significantly higher consultation rate comparing to the other age groups. The 30-day incidence rates of influenza-like illness (ILI) and acute respiratory illness (ARI) were 0.8 and 7.2% respectively, and the consultation rates among these participants were 91 and 64%. Private general practitioner clinics was the main service utilized by participants for most of the symptoms considered, especially those related to acute illness such as fever, diarrhoea and vomiting. Chinese medicine clinics were mostly likely to be visited by participants with low back pain, myalgia and fatigue. Among participants who have sought medical services, most were within 3 days of symptom onset.

**Conclusions:**

Healthcare-seeking behaviour were different by symptoms and age. Characterization of these patterns provides crucial parameters for estimating the full burden of common infectious diseases from facility-based surveillance system, for planning and allocation of healthcare resources.

## Background

Healthcare-seeking behaviour is defined as “any activity undertaken by individuals who perceived themselves to have a health problem or to be ill for purpose of finding an appropriate remedy” [1]. Healthcare-seeking behaviour includes the timing and types of healthcare service utilization and may affect population health outcomes [2]. Delayed medical attention has been shown to associate with an increased risk of unfavourable outcomes [3]. For patients with infectious diseases, delay in seeking care may also result in increased transmission risk in the community. Understanding the pattern of healthcare-seeking behaviour could help public health practitioners and policy makers to improve the healthcare system and health promotion strategies.

From a patients’ perspective, healthcare-seeking behaviour tends to be responsive to discomfort or symptoms, rather than to specific diagnosed diseases which were unknown to them before medical consultation. However, many studies examined healthcare-seeking behaviour either focused on a patient care perspective, or restricted to a specific disease related to a few limited symptoms [4–6]. In this study, we focused on healthcare-seeking behaviour specific to symptoms and syndromes, which may more realistically reflect personal responses to sickness in the general population. Such data is still limited in the literature.

A previous study in Denmark showed that for patients with any symptoms, on average < 40% of the patients actually sought healthcare service, though the proportion varied substantially by symptoms [7]. Here we reported the findings in Hong Kong which also has a well-developed healthcare system composed of both public and private sectors but with very different share in the outpatients and inpatients services: 70% of outpatient services were delivered by private sectors, whereas 90–95% of inpatient services were provided by public sectors [8]. Also, Hong Kong has its unique mixed culture, which provides and promotes both western and Chinese medicine in the healthcare system. Western medicine has been widely accepted and is the dominant medical system for a long time, but the Hong Kong Government has also actively promoted the development of Chinese medicine.

The objective of this study is to describe the characteristics of healthcare-seeking behaviour due to different symptoms and syndromes related to respiratory and gastrointestinal-related diseases, such as the proportions of patients seeking medical consultation, types of healthcare service utilized, and time from symptom onset to consultation. Data describing healthcare-seeking behaviour could characterize the utilization of the healthcare services, and facilitate risk communication during outbreaks, planning of health care resources, and interpretation of practitioner-based surveillance system.

## Methods

### Study design and study population

**Fig. 1:**
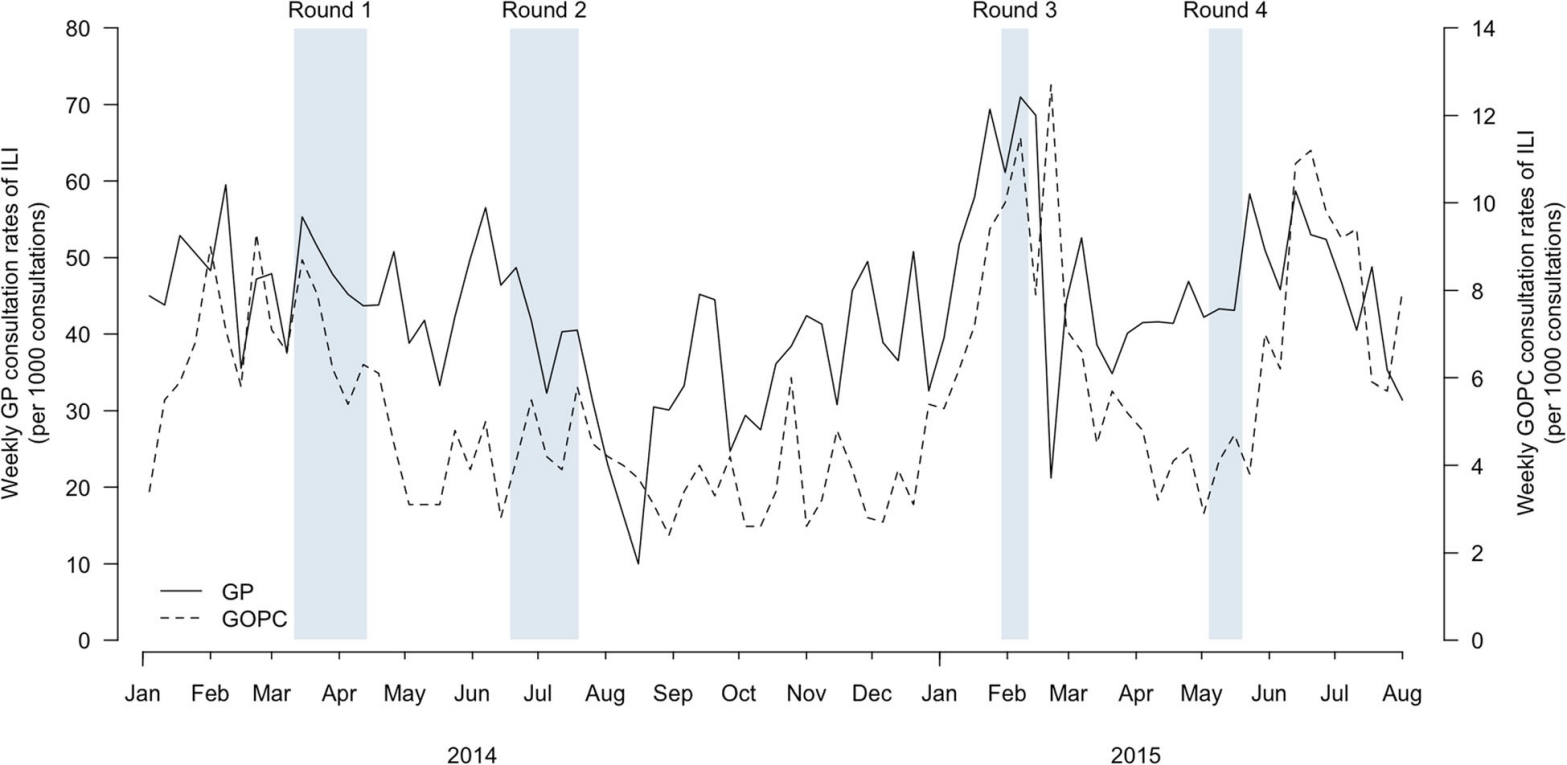
Timing of the surveys (shaded bars) and influenza-like illness consultation rates (lines) in the community from private general practitioners (GP) and public general outpatient clinics (GOPC) influenza surveillance

A longitudinal survey consisting of 4 rounds of telephone interviews was carried out from February 2014 to May 2015. We selected different times of the year to capture the variation in different infectious disease activity and also to avoid over-representation of a specific timing (e.g. winter) (Fig. 1). We avoided long holidays (e.g. Chinese New Year, Easter) which may alter typical healthcare-seeking behaviour.

The study population was the general population including children and adults in Hong Kong, a subtropical city of 7.5 million people with an ageing population of which more than 95% speak Cantonese [9]. We adopted a two-stage sampling where participants were recruited by trained interviewers through telephone calls to landlines generated by random-digit dialling. The sample size was calculated based on a previous household telephone survey, with an average of 3.5 symptoms per illness episode and a follow-up rate of about 60%,, assuming a conservative prevalence of 50% [10, 11]. Allowing for an error margin of 3% and assuming a 95% confidence level, 3000 participants would provide enough sample size to obtain accurate estimates for the top 10 symptoms. From each household, one household member aged 16 years or above was invited to participate in the study. The person who answered the phone was first recruited. To increase the sample size of the young population, we also recruited caregivers of children aged below 16 years as a booster samplevia telephone and online survey in parallel of the main survey. Up to two follow-up calls were made at different times of the day for unanswered calls. We only recruited Cantonese-speaking participants to our study. Verbal or online informed consent was obtained from participants or from parents prior to the survey. In our longitudinal study, we followed up all participants recruited in the first round and did not recruit new participants.

### Data collection

We asked the respondents about any symptoms in 30 days preceding the interview, and the corresponding healthcare-seeking behaviour. To minimize recall and reporting bias, we provided a list of 30 symptoms related to respiratory and gastrointestinal-related infections in Hong Kong [12], each of which was read out during the interview. The questionnaire was developed for this study, adopted or modified from previous questionnaires in similar studies [8, 10] (see Additional file 1). The questionnaire consisted of six main sections, including questions on [1] self-reported symptoms of the most recent illness episode [2]; healthcare-seeking behaviour (including specific symptoms leading to healthcare-seeking, types of healthcare service utilized and time from symptom onset to medical consultation) [3]; risk perception of the symptoms [4]; behavioural change and change of contact pattern due to the symptoms [5]; social-economic and host determinant of healthcare-seeking behaviour (e.g. social economic status, medical insurance, and perceived benefit of consultation) [6]; demographic information (including age, sex, education, place of living) of the participants and caregiver (if the participants are aged below 16 years). Types of healthcare service considered in our questionnaire included private general practitioner clinics (GP), general out-patient clinics (GOPC) from the public sector, Chinese medicine practitioner clinics (CMP), and Accident and Emergency Department (A&E). For the main outcome healthcare-seeking behaviour, we specifically asked the participants which symptoms directly triggered their healthcare-seeking behaviour. Besides studying healthcare-seeking behaviour by specific symptoms, we also grouped symptoms into influenza-like illness (ILI) and acute respiratory illness (ARI). ILI was defined as fever (≥37.8 °C) plus cough or sore throat; ARI was defined as any two of the symptoms including fever (≥37.8 °C), chills, headache, myalgia, cough, runny nose, and sore throat. We collected information on time from symptom onset to healthcare-seeking for each symptom that the participants have reported. We interviewed all participants irrespective of whether they had illness in the 30 days preceding the first interview, hence avoided selection of participants who were sick in the first round of interview.

### Statistical analysis

We described the healthcare-seeking behaviour triggered by specific symptoms and by ILI and ARI in all participants and by three age groups: children (0–15 years), adults (16–54 years), and the elderly (≥55 years). We defined a symptom as a trigger if the subject specifically stated that s/he sought medical consultation due to this symptom. We calculated proportion of participants seeking medical consultation by each symptom, by using the number of responses reporting medical consultation due to the symptom as numerator, and the number of episodes of each symptom as denominator. We calculated proportions of healthcare service type utilized and the distribution of the timing by each symptom, using the number of responses reporting medical consultation by each symptom as denominator. To avoid over-representation of healthcare-seeking behaviour triggered by the same symptoms for the same participants, we only included the first episode of a certain symptom for analysis. Also, healthcare-seeking behaviour was right-censored when symptom onset was close to the interview. We assumed that healthcare-seeking behaviour were fully observed for participants who had symptom onset more than 6 days before the interview, or those who have recovered at the time of interview. Participants who reported time from symptom(s) onset to medical consultation more than 30 days were regarded as missing data. Censored healthcare-seeking behaviours were imputed based on the fully observed data, with consideration of different days elapsed since symptom onset. Subjects who attended A&E were excluded when characterizing the duration from symptom onset to medical consultation.

Missing data were handled using multiple imputation with 100 sets of imputed datasets. We applied Rubin’s rules to obtain the overall estimates and 95% confidence intervals [13]. To achieve population representativeness, we applied post-stratification adjustment by age and sex according to local census data in 2014. Healthcare-seeking behaviours are described by medical consultation rate triggered by the symptom, healthcare service utilized by participants, and time from symptom onset to medical consultation. We used likelihood ratio test to assess potential age differences on healthcare-seeking behaviour using median *p*-values resulting from multiple imputation [14]. For better presentation, we combined symptoms which are related (e.g. eye problems) or having fewer than 20 reported illness episodes. All analyses were conducted in R version 3.3.3 (R Foundation for Statistical Computing, Vienna, Austria). A *p*-value of less than 0.05 was considered to be statistically significant.

## Results

### Incidence of illness and proportion of healthcare seeking

We recruited 3253 participants in the first round of survey, regardless of whether illness was reported 30 days preceding the interview, and received a total of 8727 responses throughout 4 rounds of telephone survey from February 2014 to May 2015. The response rate of the main sample was 29.0% in the first round, with follow-up rates of 73.6, 57.3 and 41.4% in rounds 2 to 4 respectively. The booster samples were recruited by referrals, with follow-up rates of 56.4, 42.0 and 22.0% from rounds 2 to 4 respectively. Among the 8727 responses, a total of 4370 illness episodes were reported from 2564 participants (Table 1), resulting in 7914 reported symptoms. The onset of 763 reported illness episodes were within 7 days of the telephone interview and for those participants who have not reported seeking medical consultations, their healthcare-seeking behaviours were considered right-censored and were handled using multiple imputation. After excluding the recurring symptoms, a total of 7120 reported symptoms from 4015 illness episodes were included for analysis (Tables 2 & 3). Symptoms related to chronic conditions usually had a larger number of repeated episodes, such as fatigue (201 recurring symptoms) and headache (118 recurring symptoms).

**Table 1:** Age-sex distribution of the study responses in Hong Kong, February 2014 to May 2015 (*N* = 3253)

Sex	Age group (y)	No. responses (%) (*n* = 8727)^b^	Hong Kong population, %^a^	Post-stratification weights
Female	0–2	95 (1.1)	1.0	0.94
	3–6	74 (0.8)	1.5	1.81
	7–15	137 (1.6)	3.2	2.06
	16–24	409 (4.7)	5.2	1.11
	25–34	742 (8.5)	8.7	1.03
	35–44	570 (6.5)	9.3	1.42
	45–54	324 (3.7)	9.3	2.52
	55–64	193 (2.2)	7.4	3.36
	65+	39 (0.4)	8.1	18.02
Male	0–2	100 (1.1)	1.1	0.96
	3–6	98 (1.1)	1.7	1.47
	7–15	96 (1.1)	3.4	3.12
	16–24	416 (4.8)	5.3	1.11
	25–34	1919 (22.0)	6.3	0.29
	35–44	1871 (21.4)	6.4	0.30
	45–54	978 (11.2)	7.7	0.69
	55–64	529 (6.1)	7.2	1.19
	65+	135 (1.5)	7.1	4.56

^a^year-end population from Hong Kong Census and Statistics Department, 2014

^b^2 female participants had missing values in age

**Table 2:** Age- and sex-standardized 30-day incidence of different symptoms or syndromes and corresponding proportion of healthcare seeking (HS) in Hong Kong, February 2014 to May 2015 (N = 3253). The symptoms were ordered descendingly based on HS %

Symptoms	No. episodes (30-day incidence rate, %)	Reported no. medical consultation	HS %^a^ (95% CI)
Fever	259 (2.1)	173	77.8 (72.7, 82.8)
Rash	25 (0.2)	13	59.8 (40.8, 78.8)
Shortness of breath	92 (0.7)	27	58.2 (46.5, 69.9)
Eye-related^b^	72 (0.6)	20	50.5 (39.6, 61.4)
Diarrhoea	203 (1.6)	87	48.7 (40.3, 57.1)
Vomiting	129 (1.0)	52	47.7 (38.7, 56.6)
Cough	643 (5.6)	239	46.5 (42.3, 50.6)
Runny nose	846 (7.7)	270	44.3 (40.1, 48.5)
Sore throat	540 (4.6)	163	44.4 (39.8, 49.0)
Headache	862 (7.7)	153	34.3 (29.8, 38.7)
Dizziness	436 (3.6)	77	34.1 (28.5, 39.7)
Chills	227 (1.8)	62	31.8 (24.2, 39.3)
Abdominal pain	162 (1.3)	38	30.4 (22.6, 38.3)
Nausea	143 (1.1)	27	29.9 (13.3, 46.6)
Loss of appetite	231 (1.8)	38	27.6 (18.6, 36.6)
Low back pain	624 (5.3)	64	23.6 (19.3, 27.8)
Myalgia	551 (4.7)	63	18.6 (14.8, 22.4)
Fatigue	988 (9.1)	62	14.0 (11.2, 16.9)
ILI	104 (0.8)	92	90.6 (85.9, 95.3)
ARI	805 (7.2)	375	64.0 (60.1, 67.8)
Any symptoms	2564 (41.4)	806	40.2 (37.0, 43.4)

*HS* healthcare seeking, *ILI* influenza-like illness, *ARI* acute respiratory illness

^a^HS %: estimate of proportion of patients visited medical consultation due to each symptom after multiple imputation

^b^Eye-related symptoms include red eyes, eye irritation, watery eyes and mucous discharge in eye. All these symptoms had small number of reported episodes and were combined for analysis

**Table 3:** Age-specific 30-day incidence of different symptoms or symptom groups and corresponding proportion of healthcare seeking (HS) in Hong Kong, February 2014 to May 2015 (N = 3253)

	0-15y (*n* = 285)		16-54y (*n* = 2661)		≥55y (*n* = 305)		
Symptom	No. episodes (30-day incidence rate, %)	HS%^b^ (95% CI)	No. episodes (30-day incidence rate, %)	HS% (95% CI)	No. episodes (30-day incidence rate, %)	HS% (95% CI)	p-value^a^
Fever	56 (5.5)	77.6 (68.1, 87.1)	186 (1.8)	68.6 (60.6, 76.5)	17 (1.4)	98.1 (94.6, 100)	< 0.001
Rash	12 (1.1)	49.8 (24.5, 75.0)	13 (0.1)	74.2 (47.7, 100)	0 (0)	–	0.405
Shortness of breath	7 (0.6)	72.3 (45.8, 98.8)	77 (0.7)	51.7 (35.0, 68.4)	8 (0.7)	64.0 (46.7, 81.4)	0.383
Eye-related^c^	8 (0.7)	38.0 (11.1, 64.9)	57 (0.5)	34.4 (20.0, 48.8)	7 (0.6)	82.5 (68.0, 96.9)	0.362
Diarrhoea	19 (1.7)	53.2 (34.3, 72.1)	174 (1.7)	47.3 (38.2, 56.5)	9 (0.7)	52.1 (24.6, 79.6)	0.301
Vomiting	14 (1.3)	59.2 (36.6, 81.7)	106 (1.0)	50.6 (38.4, 62.8)	9 (0.7)	36.9 (21.5, 52.3)	0.318
Cough	88 (9.5)	51.2 (42.8, 59.7)	508 (5.4)	45.4 (39.8, 51.0)	47 (4.2)	45.0 (37.0, 53.1)	< 0.001
Runny Nose	106 (11.9)	52.2 (43.9, 60.5)	685 (7.6)	39.8 (35.0, 44.6)	55 (4.9)	52.1 (41.8, 62.4)	< 0.001
Sore throat	30 (2.8)	40.9 (27.8, 54.1)	479 (5.1)	38.4 (33.1, 43.8)	31 (2.7)	70.4 (59.5, 81.4)	< 0.001
Headache	13 (1.2)	3.7 (0, 11.1)	765 (8.6)	31.6 (26.8, 36.4)	84 (7.9)	44.0 (35.6, 52.4)	0.002
Dizziness	4 (0.4)	12.4 (0, 36.2)	390 (4.0)	32.3 (26.2, 38.4)	41 (3.6)	39.5 (28.3, 50.7)	0.419
Chills	7 (0.6)	52.3 (20.8, 83.7)	203 (2.0)	35.2 (27.1, 43.4)	17 (1.4)	17.8 (0, 35.7)	0.017
Abdominal pain	6 (0.5)	35.1 (8.6, 61.6)	144 (1.4)	32.0 (21.9, 42.0)	11 (0.9)	26.6 (13.1, 40.1)	0.038
Nausea	2 (0.2)	39.5 (1.3, 98.7)	127 (1.2)	31.2 (20.1, 42.2)	14 (1.2)	27.9 (0, 63.6)	0.524
Loss of appetite	31 (2.9)	25.6 (13.4, 37.7)	176 (1.7)	27.7 (19.6, 35.8)	24 (2.0)	28.6 (4.2, 53.1)	0.001
Low back pain	3 (0.3)	41.0 (0.8, 90.6)	559 (5.9)	20.3 (15.7, 24.8)	62 (5.5)	30.1 (22.0, 38.2)	0.034
Myalgia	7 (0.6)	22.2 (0, 49.0)	499 (5.3)	21.5 (16.9, 26.2)	45 (4.0)	9.5 (3.7, 15.3)	0.230
Fatigue	30 (2.8)	6.5 (0, 13.8)	874 (10.0)	15.4 (11.9, 19.0)	84 (7.9)	12.2 (6.4, 17.9)	< 0.001
ILI	35 (3.3)	91.7 (84.3, 99.1)	62 (0.6)	84.0 (74.6, 93.4)	7 (0.6)	100 (59.0, 100)	0.106
ARI	93 (10.0)	75.4 (68.3, 82.4)	648 (7.1)	57.4 (52.6, 62.3)	64 (5.9)	72.6 (65.1, 80.2)	< 0.001
Any symptoms	151 (20.4)	69.9 (63.4, 76.4)	2180 (46.5)	33.2 (30.2, 36.2)	233 (30.6)	44.9 (39.3, 50.5)	< 0.001

*HS* healthcare seeking, *ILI* influenza-like illness, *ARI* acute respiratory illness

^a^Likelihood ratio test for age differences in the consultation rate

^b^HS %: estimate of proportion of patients visited medical consultation due to each symptom after multiple imputation

^c^Eye-related symptoms include red eyes, eye irritation, watery eyes and mucous discharge in eye. All these symptoms had small number of reported episodes and were combined for analysis

To achieve population representativeness, we applied post-stratification adjustment for age and sex. Young male adults were over-represented, with post-stratification weights ranging from 0.3 to 1.1, while the older population was under represented in our study, with post-stratification weights of 18.0 and 4.6 in female and male participants respectively (Table 1).

Fatigue was the most frequently reported symptom (30-day incidence = 9.1%), followed by headache (7.7%) and runny nose (7.7%) (Table 2). Fever was the strongest driver to seeking medical consultation: 77.8% of the participants having fever had sought for medical consultation, followed by rash (59.8%) and shortness of breath (58.2%) (Table 2). Symptoms related to acute illness were associated with higher medical consultation rates than those related chronic illness, such as nausea, low back pain, myalgia, and fatigue.

Over a 30-day period, almost half of the adults (aged between 16 and 54 years) reported having any symptoms (46.5%), though they are least likely to seek healthcare service when comparing to other age groups (Table 3). For children, runny nose had the highest 30-day incidence rate of 11.9%, followed by cough with incidence rate of 9.5%. For adults and the elderly, fatigue (10.0 and 7.9%, respectively) and headache (8.6 and 7.9%, respectively) were most common.

When compared across age groups, incidence rates of fever, rash, vomiting, cough, runny nose, ILI and ARI were highest in children and lowest in the elderly. Incidence rate of loss of appetite was highest in children but lowest in the 16–54 years age group (Table 3). For symptoms including headache, dizziness, chills, abdominal pain, low back pain, myalgia, and fatigue, subjects aged 16–54 years had the highest incidence rates and children had the lowest incidence rates.

Older adults who had symptoms including fever, sore throat, and headache had significantly higher consultation rates comparing to other age groups (Table 3). Children were most likely to utilize medical services, while younger adults were least likely to seek medical consultation, except when they developed rash.

### Types of healthcare service utilized

**Fig. 2:**
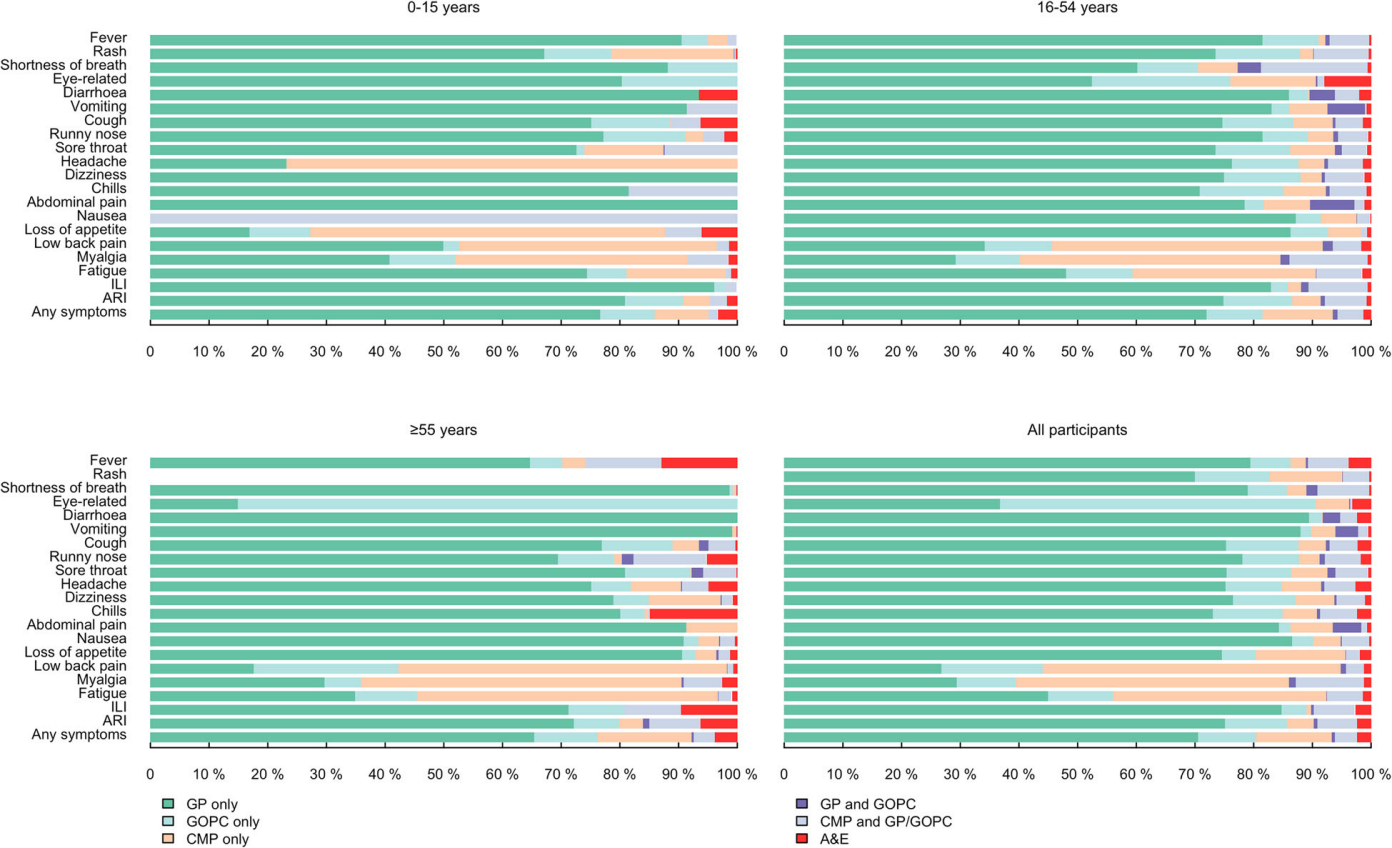
Type of healthcare services by symptoms in different age groups, among those who sought care. Healthcare services included private general practitioners (GP), public general outpatient clinics (GOPC), Chinese medicine practitioner clinics (CMP), and Accident and Emergency Department (A&E)

Regardless of specific symptoms, western medicine, i.e. GP and GOPC, was the most preferred healthcare provider, accounting for 80.9% of consultations. Private GP was the main service utilized by participants with most of the symptoms considered, especially those related to acute illness (Fig. 2). CMP was more likely to be utilized for patients with low back pain, myalgia and fatigue, and least utilized by participants with acute symptoms. 50.7% of the participants who utilized medical care due to low back pain visited CMP only. GOPC, as a public service, was only preferred by participants with eye-related symptoms, of which 53.7% visited public doctors. Considering general medical practitioners only, our study found that patients favoured GP (70.5%) over GOPC (9.9%), and relatively few participants utilized both private and public medical services (0.5%). Participants with myalgia (11.6%), shortness of breath (8.8%), and fever (7.0%) have sought both western and CMP services. 16.7% of the participants with any symptoms visited CMP, and 12.9% visiting CMP only. Most of the participants with ILI and ARI visited general medical practitioners, with proportions of 89.4 and 86.3%, respectively (Fig. 2).

Common to each age group, participants mostly consulted western medicine for acute symptoms and CMP for chronic symptoms. Children seemed more likely to consult CMP for several specific symptoms, while the other age groups consulted CMP for broader range of symptoms. Young adults were most likely to seek both western medical service and CMP, compared with other age groups. A&E visits were mostly utilized by the older population, mainly triggered by fever (12.9%), chills (14.9%), ILI (9.6%) and ARI (6.3%).

### Duration between symptom onset and medical consultation

**Fig. 3:**
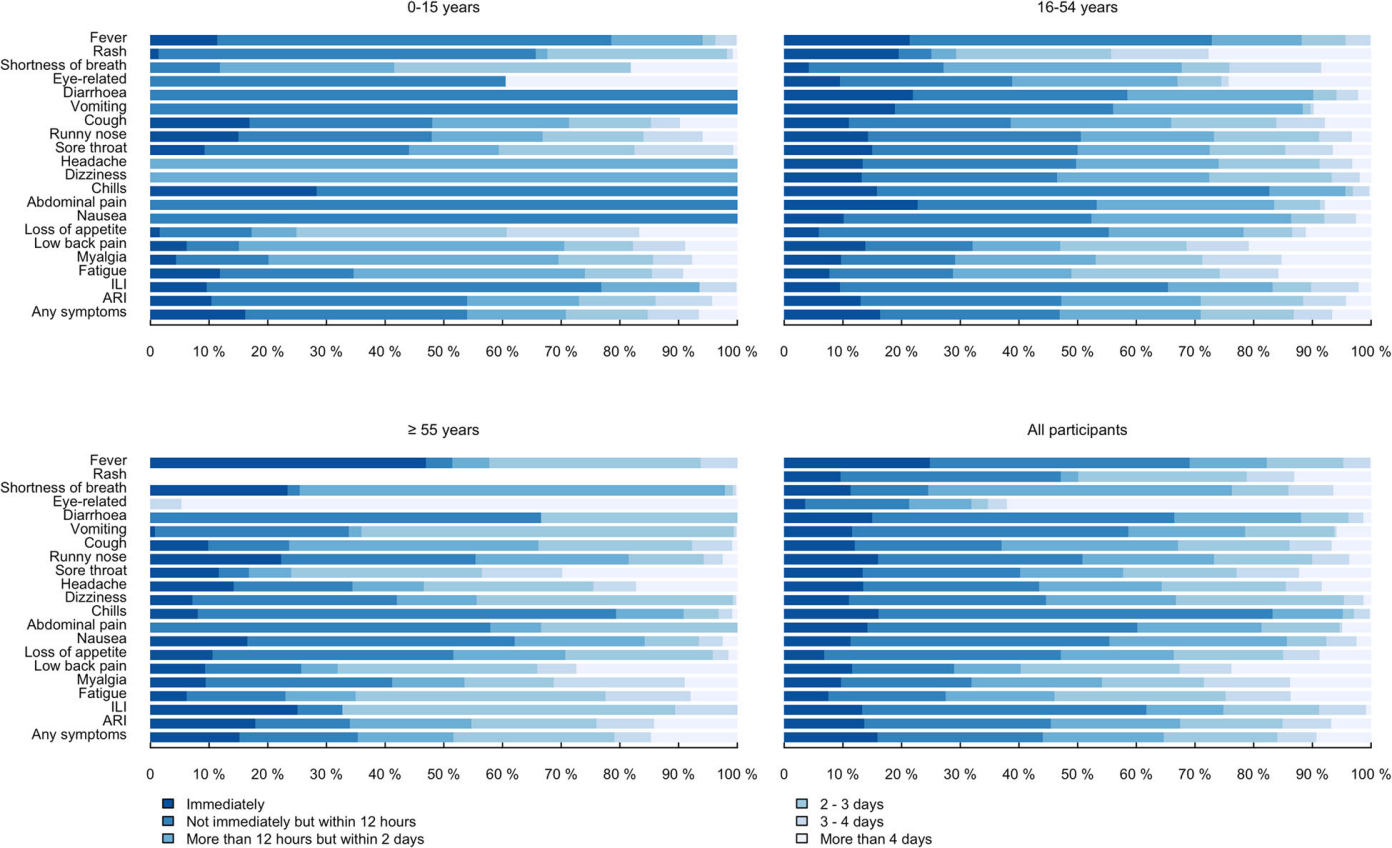
Duration from symptom onset to medical consultation for each triggering symptom, by age groups and all participants, among those who sought care. The symptoms were ordered descendingly based on proportion of seeking healthcare service

Figure 3 shows the duration between symptom onset and medical consultation in the three specific age groups and overall. Most of the participants sought medical consultation within 2 days of symptom onset regardless of symptoms. Among participants with fever, diarrhoea, vomiting, chills, abdominal pain, nausea, and ILI, more than half of the participants sought medical consultation within 12 h due to these symptoms. Among those participants who had sought medical attention due to symptoms related to acute illness and discomfort, these consultations usually took place immediately or within 12 h of symptom onsets, while it usually took longer for patients with symptoms related to chronic illness. Compared to other age groups, older participants tend to delay seeking consultation slightly. In particular, most of the older participants reported with fever either sought medical services immediately, or delay it to 2 days after symptom onset.

## Discussion

We studied healthcare-seeking behaviour specific to symptoms, which allows interpretation and application of the results in the patient perspective for Hong Kong Chinese population. Our study found that nearly half of the participants reported infectious diseases-related symptoms over a 30-day period, and 41.4% of whom have sought medical consultation (Table 2). Consultation rate varied across symptoms, ranging from 14% due to fatigue, to 78% due to fever, and was usually higher among those with acute/infectious symptoms and lower among those with mild/chronic symptoms (Table 2). The consultation rates were highest in the children and lowest in young adults, suggesting that the working population is least likely to seek medical attention when having infectious disease-related symptoms.

An overall consultation rate of about 40% (Table 2) for symptomatic patients of respiratory and gastrointestinal-related infections suggested that the majority of patients were not captured by the healthcare system, forming the submerged part of the disease iceberg. Understanding the proportions of medically unattended patients may help policy makers for developing health campaigns targeting these individuals or estimating the full burden of disease.

In Hong Kong, the private sector is the major provider of primary care, delivering about 70% of outpatient consultations [8], and CMP is used as the main alternative and complementary healthcare service in Hong Kong. In our study, we also found that western medicine is the preferred healthcare provider, contributing more than 80% of the consultations (Fig. 2). 16.7% of consultations visited CMP (Fig. 2). A local study showed that 85% of people who have sought medical consultation had consulted western medicine, while 10% had consulted CMP [8]. Another study found that 8.8% of respondents who reported symptoms during the 30 days before survey had visited a CMP for the discomfort [15]. In comparison, our finding shows that the preference for CMP may have increased slightly in the last decade with the promotion of Chinese Medicine by the Hong Kong Government. Many patients utilized both systems in parallel, taking western medicine to relieve symptoms and Chinese medicine to restore balance and health. In our study, 3.8% of participants had sought both western and Chinese medicine consultation for the same illness episode (Fig. 2). This could be interpreted as integrative medicine, or was in fact doctor shopping.

Participants had different preference on the type of health service according to their symptoms. Participants with acute symptoms favoured western medicine, whereas participants with gradually developing symptoms prefer to visit CMP. This preference could be explained by the common perception that western medicine is ‘powerful and quick’ comparing to CMP [16]. Chan et al. found that older, poorer people who have chronic conditions were more sceptical of western physicians [17]. In our study, we also found that older people having chronic symptoms such as low back pain, myalgia, and fatigue have 10–20% higher utilization of CMP than those of younger age. Considering western medicine only, our study found that patients favoured GP over GOPC regardless of their symptoms, consistent with a study showing that 76% of patients utilized primary care service provided by GPs [4].

Meng et al. [18] investigated the difference in healthcare-seeking behaviour of patients with ILI (defined as “at least two of the signs or symptoms [fever ≥37.8 °C, cough, sore throat, headache, or myalgia]”, more similar to the definition of ARI in our study) between summer and winter influenza epidemics. Meng et al. [18] found that 25.0 and 38.6% of respondents reported ILI in summer and winter peak, respectively. Among those with ILI, 42.3 and 48.5% had sought medical care for each peak, respectively. In our study 64.0% of those with ARI sought medical care (Table 2), probably because our surveys were carried out closer to the influenza peak period. In a US study, 40 and 56% of the adults and children respectively who had ILI sought healthcare service during the 2009 H1N1 pandemic [19], compared to 92 and 84% in our study (Table 2). Patients in Hong Kong were much more likely to seek medical attention when presenting with influenza-associated symptoms.

In our study, 91.7 and 75.4% of the children with ILI and ARI respectively sought medical consultation (Table 2). In Israel, 81.5% of the children under 13 year-old consulted a physician when they had flu-like symptoms [20]. Both studies showed that children with flu-related symptoms would have a high consultation rate. Age difference in the consultation rate was statistically significant only for ARI (*p*-value < 0.001) but not ILI (*p*-value = 0.106), with adults having ARI noticeably less likely to seek medical consultation (Table 3). Comparing with ARI, ILI is more specific to influenza infection, and led to high consultation rates irrespective of age (Table 3). The high consultation rates due to ILI may result in school or work absence, which probably reduced influenza transmission risk in schools or workplace. In Hong Kong, medical certificate is required for taking sick leave according to the Employment Ordinance. Though this may not be strictly enforced for short sick leave of 1 or 2 days, the need of medical certificate for the working population cannot explain the lower healthcare-seeking behaviour among adults.

Previous studies showed that some influenza patients did not visit doctors. The proportions vary across countries, for example 55% of ILI patients in the US [21], and 38% of cases of self-defined influenza in France [22]. From our study, the proportions were lower in Hong Kong (10 and 35% for patients with ILI and ARI respectively, Table 2). Most of the influenza surveillance systems are established in the clinical settings, which limits its ability to fully capture the burden of ILI/ARI for patients who have mild symptoms or do not seek any medical consultation. Our findings may help to estimate the proportion not being captured in the surveillance system.

Few studies examined the duration between symptom onset to medical consultation for common infectious diseases, in particular with respective to specific symptoms. In our study, more than 60% of participants had sought medical care within 2 days from symptom onset (Fig. 3). A US study showed that among adults with seasonal influenza, 35 and 47% sought medical care within 2 days and within 3–7 days of illness onset respectively [21], compared to our results for adults with ILI (65 and 35% respectively, combining age groups 16–54 years and ≥ 55 years in Fig. 3). The relatively short duration from illness to medical attention in Hong Kong may be attributed to easy access of medical service in a compact city. Delayed access to healthcare might be associated with longer hospital stays and poorer health outcomes [23]. Shorter duration between symptom onset and medical consultation may allow patient to have more timely diagnosis and better health outcomes.

There are a few limitations in our study. First, our data had a relatively low response rate and might suffer from under-representation of the older population. We addressed this issue by applying post-stratification weighting methods. Second, some other factors that may affect symptom-specific healthcare-seeking behaviour such as self-medication, and vaccination status were not explored in this descriptive study. Third, there may be recall bias for reporting the illness in the past 30 days. We specifically asked the participants to report the latest illness episode, and provided a list of symptoms to minimize under-reporting. However, very mild and unattended symptoms could still be missed from the survey, especially for symptoms reported by parents of younger children. Fourth, there is seasonal variation in disease activities, the associated symptoms and potentially healthcare-seeking behaviour trigged by these symptoms.

## Conclusions

Healthcare-seeking behaviour varied substantially by infectious-disease associated symptoms and age for the Hong Kong population. People with acute symptoms were more likely to see western medicine, and people with symptoms related to chronic conditions favoured Chinese medicine. Characterization of these patterns provides crucial parameters for estimating the full burden of common infectious diseases from facility-based surveillance system, for planning and allocation of healthcare resources.

## Supplementary information

**Additional file 1.** Healthcare-seeking Behavior Survey.

## Data Availability

The datasets generated and/or analysed during the current study are not publicly available but are available upon request to the corresponding author.

## Abbreviations

A&E: Accident and emergency department
ARI: Acute respiratory illness
CI: Confidence interval
CMP: Chinese medicine practitioner clinics
GOPC: General out-patient clinics
GP: General practitioner clinics
HS: Healthcare seeking
ILI: Influenza-like illness
