# Supplementary material for: A population-based study on healthcare-seeking behaviour of persons with symptoms of respiratory and gastrointestinal-related infections in Hong Kong
Source: BMC Public Health. 2020 Mar 27;20:402. doi: 10.1186/s12889-020-08555-2 (PMC7099823; doi:10.1186/s12889-020-08555-2)
Supplement: Supplementary file 1 — Additional file 1. Healthcare-seeking Behavior Survey. [file 12889_2020_8555_MOESM1_ESM.pdf]

Health-seeking Behavior Survey

Part 1 Self-rated Symptoms

SS1. Have you had any discomfort or illness in the past 30 days?
① No (go to SG1) ② Yes, please specify the illness and date: ___________________

SS2. Have you been fully recovered from discomfort or illness mentioned in SS1?
① No ② Yes

SS3. How do you perceive your health in the past 30 days?
①Excellent ②Very good ③Good ④Fair ⑤Poor

SS4. Have you had any of the following signs or symptoms during the most recent discomfort or illness episode in the past 30 days?

		No	Mild	Moderate	Severe	Don't know
a.	Fever ≥ 37.8°C (100°F) for 1 day or more	①	②	③	④	⑤
b.	Chills	①	②	③	④	⑤
c.	Headache	①	②	③	④	⑤
d.	Myalgia (aches or pain in muscles)	①	②	③	④	⑤
e.	Cough	①	②	③	④	⑤
f.	Shortness of breath	①	②	③	④	⑤
g.	Dizziness	①	②	③	④	⑤
h.	Runny nose	①	②	③	④	⑤
i.	Sore throat	①	②	③	④	⑤
j.	Diarrhoea (watery)	①	②	③	④	⑤
k.	Diarrhoea (bloody)	①	②	③	④	⑤
l.	Nausea	①	②	③	④	⑤
m.	Vomiting	①	②	③	④	⑤
n.	Low back pain	①	②	③	④	⑤
o.	Fatique	①	②	③	④	⑤
p.	Loss of appetite	①	②	③	④	⑤
q.	Involuntary muscle twitch	①	②	③	④	⑤
r.	Skin rash (hand / foot)	①	②	③	④	⑤
s.	Skin rash (other than hand / foot)	①	②	③	④	⑤
t.	Blister (oral cavity / tongue / palate)	①	②	③	④	⑤
u.	Blister (skin)	①	②	③	④	⑤
v.	Night sweat	①	②	③	④	⑤
w.	Bright red tongue	①	②	③	④	⑤

x.	Abdominal pain	①	②	③	④	⑤
y.	Chest pain	①	②	③	④	⑤
z.	Red eyes	①	②	③	④	⑤
aa.	Eye irritation	①	②	③	④	⑤
ab.	Watery eyes	①	②	③	④	⑤
ac.	Swollen glands (around ear / neck)	①	②	③	④	⑤
ad.	Mucous discharge in eye	①	②	③	④	⑤
ae.	Other, please specify: __________________	①	②	③	④	⑤

SS6. Do you have any doctor-diagnosed chronic diseases?
①No ②Yes, please specify: ___________________ .

SS7. Are you on regular medication?
①No ②Yes

Part 2 Health Services Utilization

HS1. Have you used any of the following health services due to any of the above symptoms:

		Yes		No
a.	Private hospital/ doctor (including 24hours clinics/A&E services) ("GP")	☐	______ times	☐
b.	Department of Health General Out-Patient Clinic ("GOPC")	☐	______ times	☐
c.	Chinese medicine (medical/general/orthopaedic/acupuncture) ("CMP")	☐	______ times	☐
d.	Public Hospital A&E Services ("A&E")	☐	______ times	☐

HS2. Which symptoms prompt you to seek for medical consultation?
HS3. How long is the time from symptom onset to medical consultation (for each selected symptoms in SS2)?

	Symptoms	Prompt for consultation	Medical Services	Time from symptom onset to consultation	Self-medication
a.	Fever ≥ 37.8°C (100°F) for 1 day or more	Yes / No	GP / GOPC / CMP / A&E	Immediate / within 12 hours / __days	Yes / No
b.	Chills	Yes / No	GP / GOPC / CMP / A&E	Immediate / within 12 hours / __days	Yes / No
c.	Headache	Yes / No	GP / GOPC / CMP / A&E	Immediate / within 12 hours / __days	Yes / No
d.	Myalgia (aches or pain	Yes / No	GP / GOPC / CMP / A&E	Immediate / within 12 hours / __days	Yes / No

	in muscles)				
e.	Cough	Yes / No	GP / GOPC / CMP / A&E	Immediate / within 12 hours / __days	Yes / No
f.	Shortness of breath	Yes / No	GP / GOPC / CMP / A&E	Immediate / within 12 hours / __days	Yes / No
g.	Dizziness	Yes / No	GP / GOPC / CMP / A&E	Immediate / within 12 hours / __days	Yes / No
h.	Runny nose	Yes / No	GP / GOPC / CMP / A&E	Immediate / within 12 hours / __days	Yes / No
i.	Sore throat	Yes / No	GP / GOPC / CMP / A&E	Immediate / within 12 hours / __days	Yes / No
j.	Diarrhoea (watery)	Yes / No	GP / GOPC / CMP / A&E	Immediate / within 12 hours / __days	Yes / No
k.	Diarrhoea (bloody)	Yes / No	GP / GOPC / CMP / A&E	Immediate / within 12 hours / __days	Yes / No
l.	Nausea	Yes / No	GP / GOPC / CMP / A&E	Immediate / within 12 hours / __days	Yes / No
m.	Vomiting	Yes / No	GP / GOPC / CMP / A&E	Immediate / within 12 hours / __days	Yes / No
n.	Low back pain	Yes / No	GP / GOPC / CMP / A&E	Immediate / within 12 hours / __days	Yes / No
o.	Fatique	Yes / No	GP / GOPC / CMP / A&E	Immediate / within 12 hours / __days	Yes / No
p.	Loss of appetite	Yes / No	GP / GOPC / CMP / A&E	Immediate / within 12 hours / __days	Yes / No
q.	Involuntary muscle twitch	Yes / No	GP / GOPC / CMP / A&E	Immediate / within 12 hours / __days	Yes / No
r.	Skin rash (hand / foot)	Yes / No	GP / GOPC / CMP / A&E	Immediate / within 12 hours / __days	Yes / No
s.	Skin rash (other than hand / foot)	Yes / No	GP / GOPC / CMP / A&E	Immediate / within 12 hours / __days	Yes / No
t.	Blister (oral cavity / tongue / palate)	Yes / No	GP / GOPC / CMP / A&E	Immediate / within 12 hours / __days	Yes / No
u.	Blister (skin)	Yes / No	GP / GOPC / CMP / A&E	Immediate / within 12 hours / __days	Yes / No
v.	Night sweat	Yes / No	GP / GOPC / CMP / A&E	Immediate / within 12 hours / __days	Yes / No
w.	Bright red tongue	Yes / No	GP / GOPC / CMP / A&E	Immediate / within 12 hours / __days	Yes / No
x.	Abdominal pain	Yes / No	GP / GOPC / CMP / A&E	Immediate / within 12 hours / __days	Yes / No
y.	Chest pain	Yes / No	GP / GOPC / CMP / A&E	Immediate / within 12 hours / __days	Yes / No
z.	Red eyes	Yes / No	GP / GOPC / CMP / A&E	Immediate / within 12 hours / __days	Yes / No
aa.	Eye irritation	Yes / No	GP / GOPC / CMP / A&E	Immediate / within 12 hours / __days	Yes / No
ab.	Watery eyes	Yes / No	GP / GOPC / CMP / A&E	Immediate / within 12 hours / __days	Yes / No
ac.	Swollen glands (around ear / neck)	Yes / No	GP / GOPC / CMP / A&E	Immediate / within 12 hours / __days	Yes / No
ad.	Mucous discharge in eye	Yes / No	GP / GOPC / CMP / A&E	Immediate / within 12 hours / __days	Yes / No
ae.	Other, please specify: ________________	Yes / No	GP / GOPC / CMP / A&E	Immediate / within 12 hours / __days	Yes / No

HS4. (If HS1=Yes) What is the diagnosis from your selected medical service(s)?

a. Private hospital/ doctor (including 24hours clinics/A&E services) (“GP”) ________________

b. Chinese medicine (medical/general/orthopaedic/acupuncture) ("CMP") ________________

c. Department of Health General Out-Patient Clinic ("GOPC") ________________

d. Public Hospital A&E Services ("A&E") ________________

Part 3 Other Information on Medical Consultation

MC1. Are you holding any medical benefits or insurance?
①No ②Yes, employer-provided medical benefits ③ Yes, private medical insurance ④ Yes, both

MC2. Do you consider seeking of medical consultation significantly affect your normal life?
① Never ② Not at all ③ Sometimes ④ Moderately So ⑤Very Much So

MC3. Do you consider seeking medical consultation helpful to recovery?
① Never② Not at all ③ Sometimes ④ Moderately So ⑤Very Much So

MC4. Do you consider you may handle the symptoms well by yourself?
① Never② Not at all ③ Sometimes ④ Moderately So ⑤Very Much So

Part 4 Demographics

SG1. Gender
①Female ②Male

SG2. What is your (or target respondent) age?
a) _____years
b) (1) 16-24 (2) 25-34 (3) 35-44 (4) 45-54 (5) 55-64 (6) 65 or above (7) Refused

SG2b. What is the target respondent's age (If caregiver was interviewed)?
a) _____years
b) (1) 0-2 (2) 3-6 (3) 7-15 (4) Refused

SG3. What is your place of birth?
①Hong Kong ②Guang Dong province ③Macau ④Other provinces of China
⑤Other _____
*(If ②-⑤, How many years have you lived in Hong Kong? ____)

SG4. What is your marital status?
①Single ②Married ③Divorced /separated ④Widowed

SG5. How many people live in your household, including yourself and domestic helper(s)? ___

SG6. What is your education level?
①no schooling/kindergarten ②Primary 1 to 3 ③Primary 4 to 6 ④Secondary 1 to 3
⑤Secondary 4 to 5 ⑥Matriculation ⑦Tertiary or above

SG7. What is your occupation?
(01) Retired (02) No job, unemployed, seeking job (03) Full-time students (04) Home makers
(05) Professionals (06) Associate professionals (07) Clerks (08) Service workers and shop sales workers
(09) Skilled agricultural and fishery workers; and occupations not classified (10) Craft and related workers
(11) Plant and machine operators and assemblers (12) Elementary occupations (13) Managers and administrators

SG8. Where do you live?
(01) Central & Western (02) Southern (03) Eastern (04) North (05) Wan Chai (06) Sham Shui po (07) Yuen Long
(08) Tai Po (09) Yau Tsim Mong (10) Tsuen Wan (11) Wong Tai Sin (12) Sha Tin (13) Kwun Tong (14) Kowloon City
(15) Islands (16) Sai Kung (17) Kwai Tsing (18) Tuen Mun

SG9. Do you have any family members with chronic illnesses?
①No ②Yes, please specify: ___________________________

SG10. Did you travel abroad in the last 30 days?
a) ①Yes ②No
b) If "Yes", please specify:___________________________
c) When did you travel back to Hong Kong: ___(month) ___(day) (or ___days before)

SG11. What is your monthly household income:

(1) < $5,000	(2) $5,000 - $9999
(3) $10,000 - $14,999	(4) $15,000 - $19,999
(5) $20,000 - $24,999	(6) $25,000 - $29,999
(7) $30,000 - $34,999	(8) $35,000 - $39,999
(9) ≥ $40,000	(10) No income

SG12. Would it be possible to contact you again three months later for a follow-up survey?
①Yes ②No

SG13. Name / identifier (Chinese): _______________________ *(Needed if SG12 = Yes, optional if SG12 = No)
